# Development of a web-based tool to assess daily rating of perceived exertion in agility dogs

**DOI:** 10.3389/fvets.2024.1473977

**Published:** 2024-12-06

**Authors:** Debra C. Sellon, Abigail Shoben, Arielle Pechette Markley, Dianne McFarlane, Denis J. Marcellin-Little

**Affiliations:** ^1^Department of Veterinary Clinical Sciences, College of Veterinary Medicine, Washington State University, Pullman, WA, United States; ^2^Division of Biostatistics, College of Public Health, The Ohio State University, Columbus, OH, United States; ^3^Department of Veterinary Clinical Sciences, College of Veterinary Medicine, The Ohio State University, Columbus, OH, United States; ^4^Department of Large Animal Clinical Sciences, College of Veterinary Medicine, University of Florida, Gainesville, FL, United States; ^5^Department of Surgical and Radiological Sciences, School of Veterinary Medicine, University of California, Davis, Davis, CA, United States

**Keywords:** agility, dog, rating of perceived exertion, sports medicine, training load

## Abstract

**Objective:**

To develop a web-based tool for daily use by agility handlers to log rating of perceived exertion (RPE) for dogs as an aid in quantifying daily exercise and training load and to improve training and conditioning strategies.

**Procedures:**

Focus group meetings with small groups of handlers were conducted via internet—based video conferencing using a semi-structured interview format. Meeting notes were coded for reflexive thematic analysis. The RPE logging tool was revised based on handler feedback. Each handler was asked to log their dog’s daily RPE data for 1 week. Data were analyzed to assess compliance and timeliness of entries. Participants completed a post-logging questionnaire to provide feedback about their experiences.

**Results:**

Eighteen agility dog handlers participated in all phases of the project. Handler and dog demographics were similar to previously reported demographics of agility participants in the United States. Reflexive thematic analysis of their comments related to the initial draft RPE logging tool yielded 3 initial themes, all of which supported a fourth and major theme: the need for specific and detailed training resources before agility handlers utilized the RPE tool. Of 18 handlers, 14 (78%) submitted logging records for the full week. Median time for data entry was 87 s (IQR = 56–117 s), and 92% of logging records were entered within 1 day of the events which were being recorded. In the follow-up questionnaire the handlers did not identify any major concerns. Based on all available data from the handlers, a final version of the RPE logging tool was produced.

**Conclusion and clinical relevance:**

Agility dog handlers are very interested in developing and validating tools to quantify training load for their dogs. The final RPE logging tool was quick and easy to use. Further validation of this logging tool is required with a larger number of handlers and comparison to physiologic data from exercising dogs.

## Introduction

Canine agility is growing in popularity, with a concomitant increase in interest in evidence-based practices that support optimal athletic performance and competitive longevity. The sport of agility is physically demanding because it combines running, jumping obstacles, frequent abrupt turns at speed, navigation of elevated and angled frames or teeter-totters, and weaving between tightly spaced poles. Retrospective studies of agility dog injuries based on handler reports estimate that one-third or more of agility dogs experience one or more injuries in their competitive career, with one-third of those dogs having more than one injury (1–8). The most common anatomic sites reported to be injured are the shoulder, back, neck, and digits (2–6, 9, 10).

A recent survey of more than 1,300 agility handlers ranked the relative importance of 12 research areas related to canine agility (10). The highest ranked research priorities were enhancing and prolonging the athletic lifespan for dogs, identifying risk factors for specific types of injuries, physical conditioning programs, rehabilitation programs for injured dogs, improving safety of equipment and course design, and understanding safety of various surfaces used of agility training and competition. Each of these areas of research would benefit from the ability to collect data related to canine training and activity load in an accurate, efficient, and prospective manner.

Training and competition load in human athletes refers to the total volume, intensity, and type of physical activity undertaken by the athlete over a period of time (11). This concept includes both external training load, what the athlete does, and internal training load, the psychobiological responses to these activities (11). The internal training load experienced as a result of the work performed (external training load) can change according to fitness status of the athlete. External load (the physical work executed) and internal load (the biological response) can now be simultaneously measured in many ways in human athletes such as Global Positioning System (GPS) monitoring combined with heart rate monitors (12). Training and competition load stimulates adaptation of body systems which can result in increased fitness and improved performance. There are currently no validated tools to measure daily athletic activities and “training load” of agility dogs. There are only a few reports of potential links between activity, conditioning, or training practices and risk of injury in agility dogs (3, 13). In contrast, there is an abundance of information on this topic related to human athletes in a wide variety of sports (14–17) and load management has emerged as an important factor in injury risk (14). Objective exercise data are also used to study factors predisposing racing horses to injury (18, 19).

Training or sport exposure can be recorded in a variety of ways, including daily training logs, activity monitoring with electronic devices, recording of specific event frequencies and durations, and self-report ratings of perceived exertion (20). The rating of perceived exertion (RPE) as reported by the athlete after each training session was first described by Borg (21). This simple, subjective measure has been modified in numerous ways to fit athletes in multiple sports (15–17, 22–24). Despite its simplicity, the RPE and its modifications have often been more valuable in monitoring training load than objective parameters such as training days, training volume, or repetitions of individual training events. The RPE has been validated for many sports and activities, and it does not require any technology for implementation.

Assessments of RPE in children performing treadmill exercise, provided by trained external observers, corresponded with objective measures of exercise intensity and with the self-rating provided by the children (25, 26). A perceived exertion scale (0 to 4) has been described for dogs exercising on a treadmill; observer scores correlated well with objective physiologic measures (27). An RPE of 1 to 10 as assessed by trainers and riders correlated with physiologic variables of exercise intensity during race horse training sessions (28). Given that self-reported measures of training exposure are considered generally accurate for human athletes (29, 30), and that external observers provide valid ratings of exertion for children, dogs, and horses, it is reasonable to expect that handler-reported RPE would be valid as an aid in assessing training and activity load for agility dogs. The goals of this project were to develop a concise, easy-to-use RPE tool to aid in quantifying daily exercise and training load in dogs and to test its performance in a small group of agility handlers.

## Materials and methods

### Participants

Participants, referred to as “handlers” in this report, were recruited through advertisement on social media sites that targeted active agility competitors in the United States. Handlers were required to be 18 years of age or older, reside in the United States, and be currently competing (within the past 3 months) in agility with one dog or more. Handler participants were asked to complete 5 activities; (1) respond to an online enrollment questionnaire; (2) review background information introducing the concept of RPE and a draft RPE instrument for agility dogs; (3) participate in an online virtual focus group session in a semi-structured interview format; (4) use a revised draft RPE tool for 1 week; and (5) complete an online questionnaire to provide feedback about their RPE logging experience. The Institutional Review Board of Washington State University determined this project satisfied the criteria for exempt research. Anonymized survey responses and datasets generated and/or analyzed for this report are available upon reasonable request to the authors.

### Enrollment questionnaire

An internet-based questionnaire for handlers was designed on a commercial internet survey site (Qualtrics, Provo, UT).^1^ The enrollment questionnaire was adapted from previous surveys of agility handlers and consisted of 3 sections; (1) determination of eligibility for participation; (2) demographic and agility-related information about the specific dog nominated for participation; and (3) demographic information about the handler. Full text of the enrollment questionnaire is available as Supplementary Item 1.

Section 2 of the enrollment questionnaire sought to determine dog-related information including signalment (age, sex, breed), body characteristics (weight, body condition, height in inches measured at the withers), and prior involvement and experiences in agility. Agility-related questions included most frequent competition venue, highest level of agility, approximate average speed in yards per second (yps), experience at a national championship event, most common jump height, access to training facilities, and anticipated approximate number of days of competition in the next year.

Handler-related information collected in section 3 included number of dogs currently competing or training to compete in agility, number of dogs with which the handler has competed in agility over their lifetime, number of years the handler has been active in agility, types of participation in agility, medical education or training, age, and gender.

### Draft RPE logging tool

**Figure 1:**
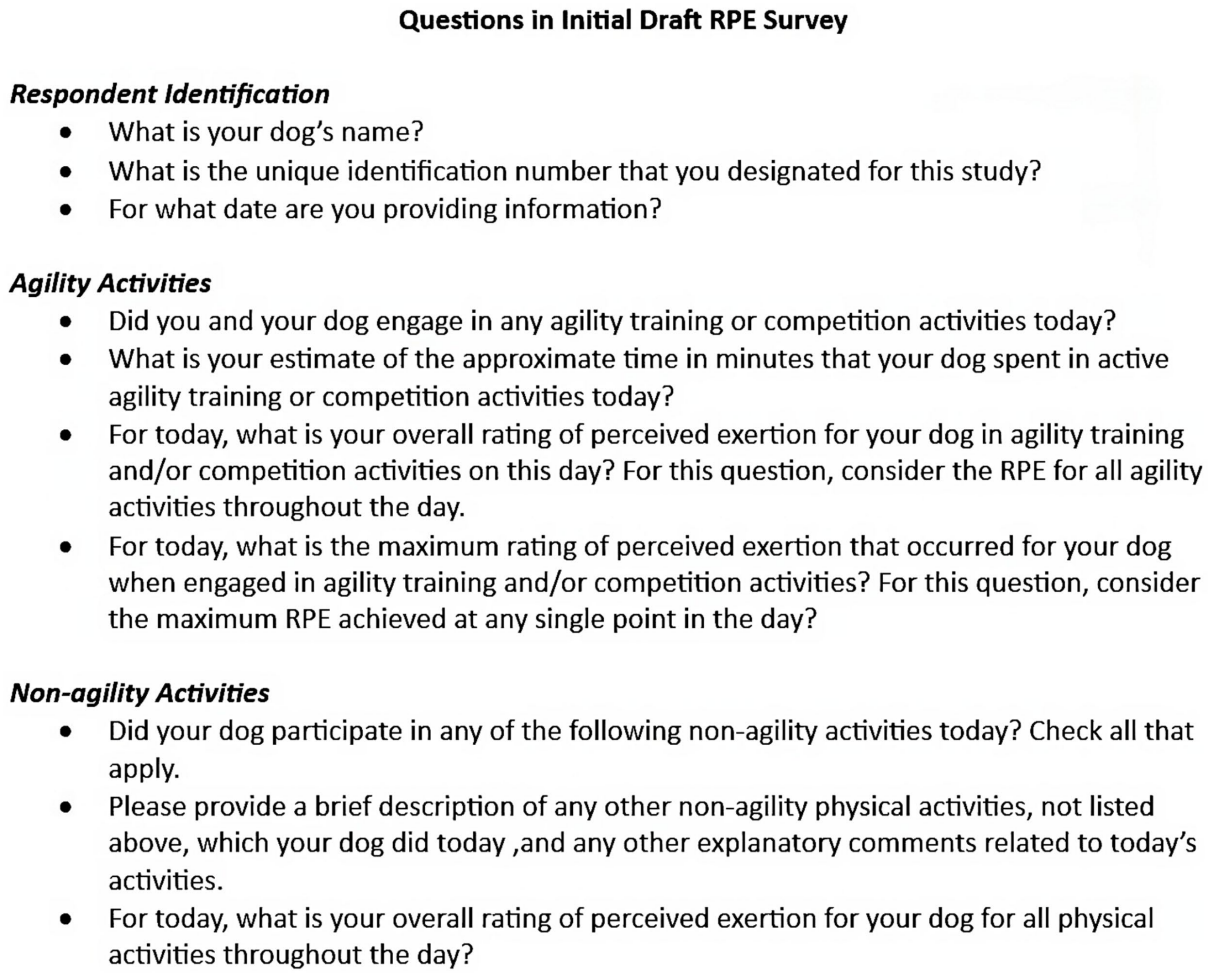
Draft RPE logging tool as initially prepared by the research team. These questions were shared with focus group participants and discussed in semi-structured interview format.

An initial draft of a daily RPE tool was prepared by consensus collaboration of the authors (Figure 1). The draft RPE was designed with the goal of optimizing quality of data collected from the participating handlers while maintaining ease of use and minimizing daily time requirements. This logging tool was developed using the same commercial internet survey site used for the enrollment questionnaire. The draft logging tool began with a section containing 3 questions intended to establish and confirm participant identification (handler name, dog name, and personal identification number [PIN]).

The main portion of the daily RPE tool contained 7 questions related to the activities of the dog on that date. Respondents were asked whether they had participated in any agility training and/or competition activities. Training or competition time was defined as the time spent training or performing in any activities related to agility, with or without using specific agility equipment or obstacles. If the respondent indicated they had participated in agility activities, they were asked to estimate the time in minutes for all agility-related activities on that date. The next question asked the handler to provide an overall RPE for all agility activities on that date in which a rating of 1 indicated no exertion at all and a rating of 10 indicated the maximal possible exertion. This was followed by a request for a separate RPE that represented the maximum RPE that occurred at any single point in time on that date. The respondent was next asked whether their dog had participated in non-agility related physical activities on that date. A list of various types of physical activities, adapted from a previous agility-related survey, was provided followed by a free text response box in which other activities or explanations could be provided. The final question asked the handler to provide an estimate of the total RPE for the dog for all activities (agility and non-agility) for that date with a rating of 1 indicating no exertion at all and a rating of 10 indicating the maximal possible exertion for the day.

### Focus groups (semi-structured interviews)

Five structured interviews were conducted using internet-based video conferencing software (Zoom Video Communications, Inc)^2^ with a maximum of 7 participants in any one session. Prior to the meeting, participants were provided with an opportunity to review the initial draft RPE logging tool. Each meeting was conducted using a detailed script with visual aids which were presented using shared screen technology. Meetings began with a review of background information, project personnel, funding, goals, eligibility criteria, methods, anticipated time commitment, and a statement of risks and benefits for participants. Participants were asked to respond to questions related to the enrollment questionnaire, which was completed by each handler prior to the focus group sessions, and the clarity of questions within the draft daily RPE logging tool. Sessions were not recorded; detailed notes of the discussion were chronicled by the investigators.

Data from the focus group interviews were analyzed using the six-phase process of reflexive thematic analysis (RTA), as described by Braun and Clarke (31). The underlying research goal for this analysis was to identify possible modifications to the daily RPE logging tool that would make it more understandable and usable by an average agility handler. Because focus group discussions were not recorded, initial coding of data was based on the investigator’s contemporaneous notes. Each note was individually assigned one or more content codes. Related codes were grouped into themes and subthemes through an iterative process. After review, themes and subthemes were used to form a thematic “map” of the analysis. Themes were ultimately defined and named. Final themes were reviewed by the research team as a whole, which included individuals with deep knowledge of agility and others with more superficial knowledge. On the basis of this analysis, a revised draft RPE logging tool was prepared.

### RPE logging

After the focus group sessions, handlers were asked to log their dog’s activities using the revised draft RPE logging tool daily for at least 7 days. An automated email reminder containing a link to the logging tool was sent to each participating handler at 12 pm (noon) Pacific standard time each day between 12/6/2023 and 12/14/2023. Date and time of data entry by each handler was automatically recorded by the survey software (date of entry). This date of entry was compared to the date of the activity which the handler indicated at the beginning of each record.

### Follow-up questionnaire

**Figure 2:**
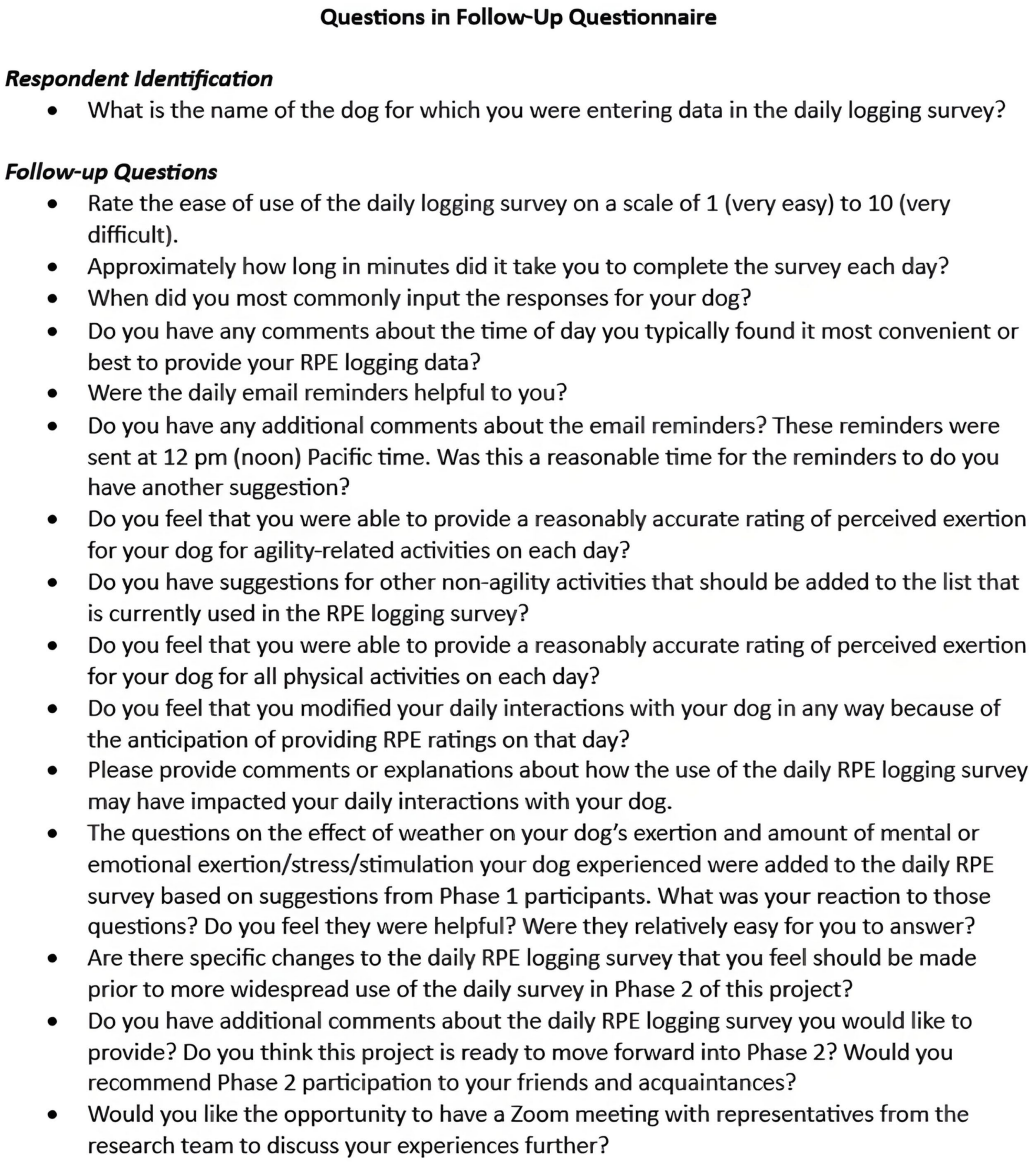
Questions included in the follow-up questionnaire for 18 handlers who provided daily RPE logging records of their dogs’ daily activities.

After logging was complete, participants were asked to complete an online questionnaire designed by the research team using the same commercial internet survey site used for enrollment and daily logging questionnaires. This questionnaire included 16 questions, most of which were open-ended. A summary of questions is shown in Figure 2 and full text of this questionnaire is available as Supplementary Item 2.

## Results

### Enrollment questionnaire

Between 10/18/2023 and 10/26/2023, 45 individuals accessed the online enrollment questionnaire. Twelve respondents (27%) did not provide personal contact information and were excluded from participation. For the remaining 33 respondents, median time for questionnaire completion was 531 s (IQR = 391–732 s). These respondents were contacted via email and provided with a list of available times for focus group discussions. Of these 33 handlers, 18 were able to schedule and participate in a focus group discussion scheduled between 10/26/2023 and 11/1/2023 (Table 1).

**Table 1:** Focus group dates and participants for discussions of the initial draft of a daily rate of perceived exertion tool.

Focus group number	Date	Handler participants	Project personnel participants
1	10/30/2023	7	3
2	11/6/2023	3	1
3	11/8/2023	2	1
4	11/14/2023	5	1
5	11/16/2023	1	1

Enrollment questionnaires of the 18 handlers who participated in a focus group discussion were further reviewed. These handlers were from 12 states. Six individuals were from Washington State, two from New York and one each from Arkansas, California, Florida, Idaho, Kentucky, Oklahoma, Oregon, Pennsylvania, Tennessee, and Virginia. Of the 17 handlers who reported their age, similar numbers of respondents were between 18 and 40 years of age (*n* = 8, 47%) and greater than 41 years of age (*n* = 9, 53%). Nearly all handlers (17/18, 94%) indicated that they were female. The number of dogs owned by each handler varied with 6 handlers (33%) owning only 1 dog, 5 handlers (28%) owning 2 dogs, 6 handlers (33%) owning 3 dogs, and 1 handler (6%) owning 4 or more dogs. Years of experience in agility varied from <3 years (4 handlers, 22%) to >15 years (4 handlers, 22%).

Ten handlers (56%) had competed in at least one national agility competition within the past 5 years. Of the 18 enrolled handlers, the preferred agility competition venue was American Kennel Club for 9 handlers (50%), Canine Performance Events for 4 handlers (22%), North American Dog Agility Council for 2 handlers (11%), United Kingdom Agility International for 2 handlers (11%), and United State Dog Agility Association for 1 handler (6%). Handlers indicated that their most common type of agility training was either regular in-person group classes with an instructor (12 handlers, 67%) or training alone at their own home or premises (5 handlers, 28%). One handler indicated that they primarily trained alone at a premises owned by another person.

There were 12 breeds of dogs represented including 5 border collies, 2 Australian shepherds, 2 Doberman pinschers, and 1 each of 9 other breeds. Mean body weight for enrolled dogs was 19 ± 8 kg (41 ± 18.4 lbs). Mean height at the withers for enrolled dogs was 19.2 ± 5.0 inches. Competition jump heights varied from 8 inches (4 dogs, 22.2%) to 24 inches (2 dogs, 11.1%) with the largest number of dogs jumping 20 inches (8 dogs, 44.4%). The highest level of competition achieved by enrolled dogs ranged from Starters/Novice/Beginner (5 dogs, 27.8%) to Masters/Elite/Excellent (11 dogs, 61.1%).

### Reflexive thematic analysis

**Figure 3:**
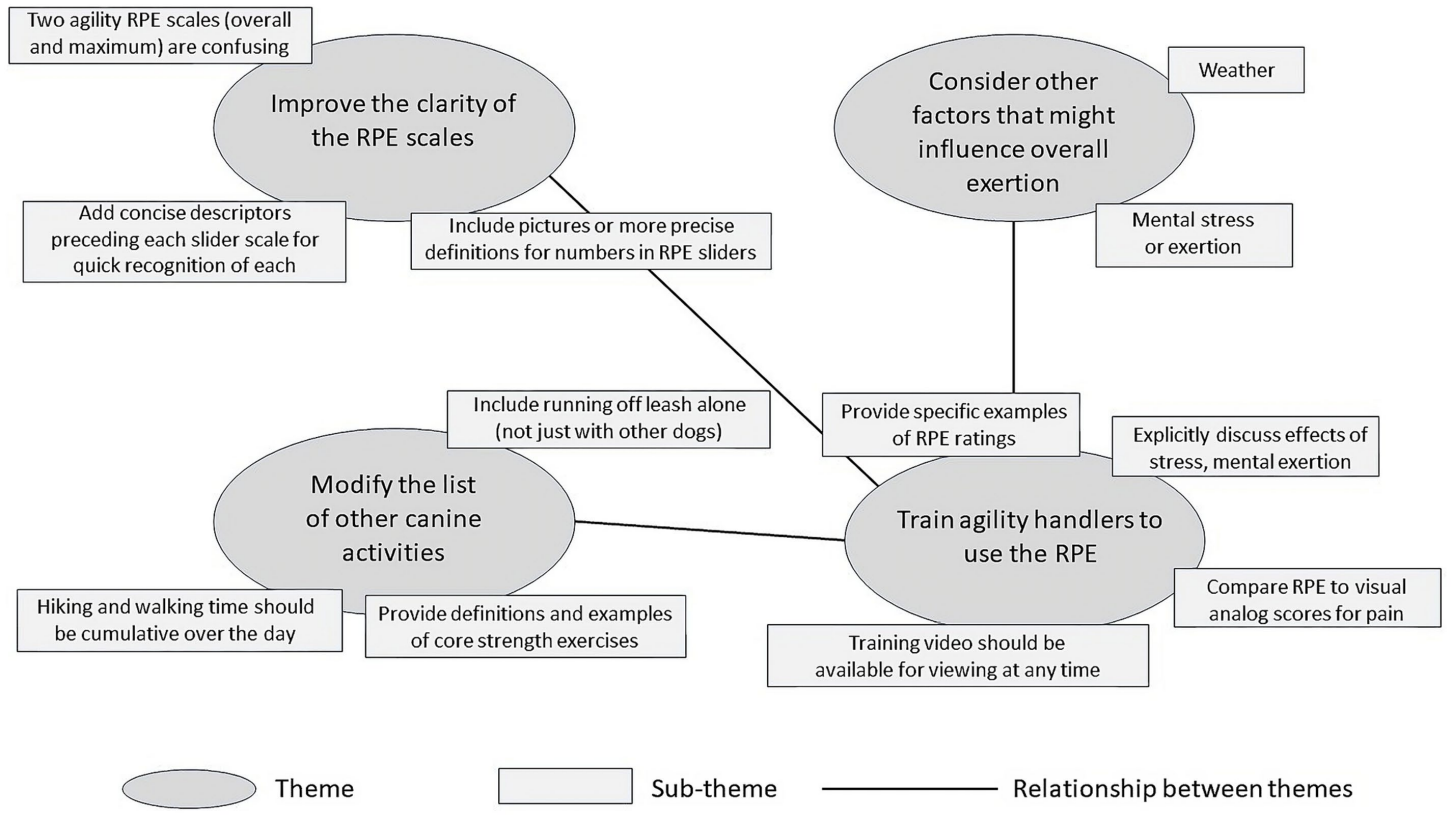
Thematic map including themes, sub-themes, and relationship between themes developed using reflexive thematic analysis of data from semi-structured interviews conducted in electronic focus group discussions related to the structure, function, and clarity of the draft RPE logging tool shown in Figure 1.

Data from focus group interviews were separated into 61 comments or questions derived from the investigators’ contemporaneous notes. Comments unrelated to the central research goal of identifying necessary modifications or clarifications to the daily RPE logging tool were excluded (*n* = 12). The remaining 49 comments and suggestions were collated into 4 themes each of which comprised two or more sub-themes (Figure 3). The most compelling theme identified was the need to modify and clarify the underlying definitions and utilization of the RPE scales. Several handlers were confused by the distinctions between the two RPE scales related to agility activities. One scale attempted to quantify the maximum agility-related exertion experienced at any single point in time on a given day; the second scale attempted to quantify the overall or cumulative level of agility-related exertion experienced by the dog on that day. Other comments requested visual or verbal descriptors on the sliding scale to assist them in conceptualizing the level of exertion associated with each number. The second theme identified in the analysis related to modifications to the list of non-agility activities in which the dogs might participate on any given day. The concerns primarily related to definitions of running, playing, hiking, and walking. There were also requests for clarification of the definitions of core strengthening and balance training and trick training. The third theme related to the ways in which weather conditions and mental stress might impact total exertion by the dog on any given day. These three themes were strongly related to the fourth theme: the need for detailed and readily available training resources. The suggestion of a training video was strongly supported, with requests that such a video include specific examples and clear definitions. There was a strong consensus that the training video should be available online so that it could be watched independently and supplemented by a mechanism to ask questions as needed.

### Revision of the RPE logging tool

**Figure 4:**
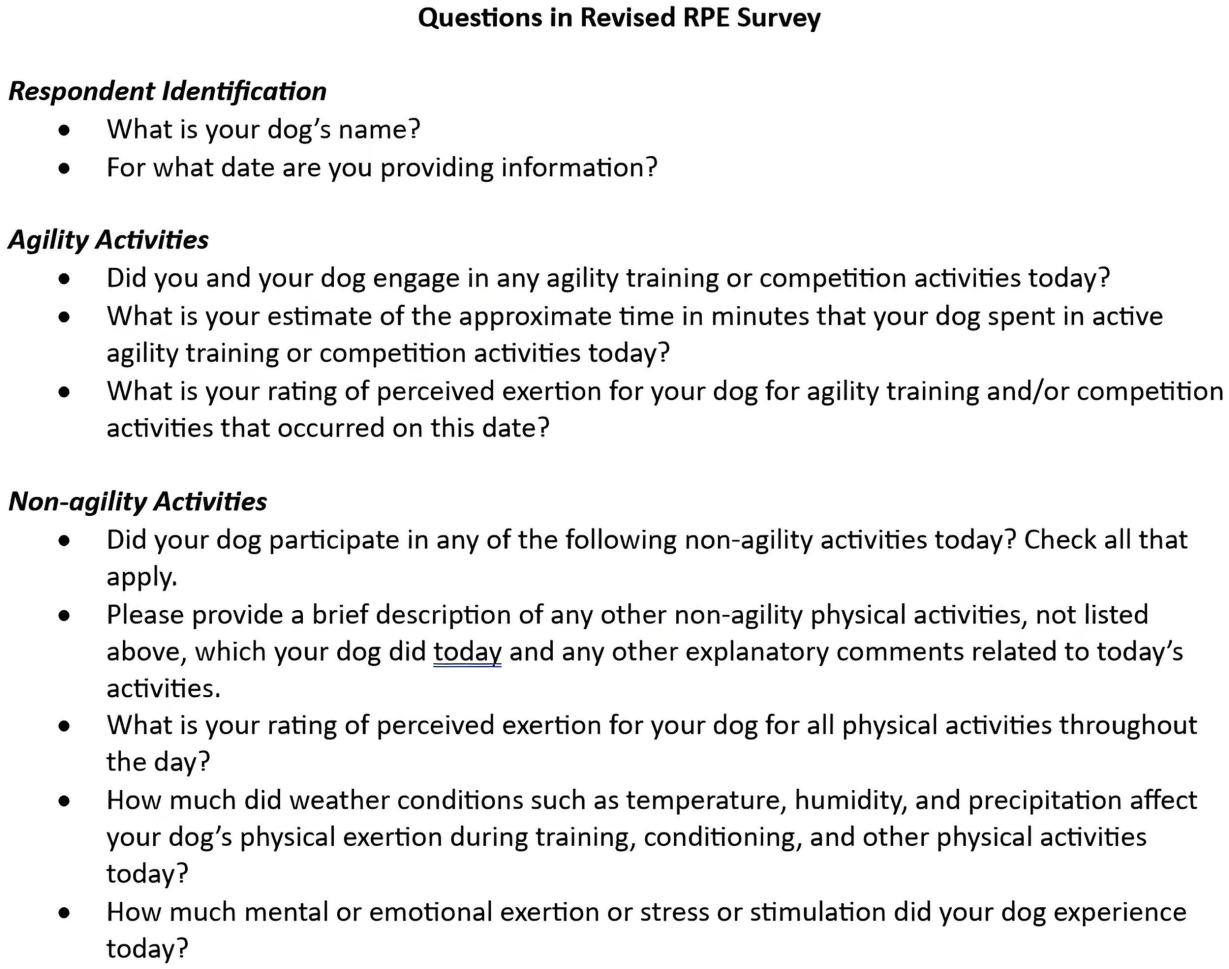
Revised draft daily RPE logging tool with changes implemented on the basis of comments from focus group interviews. This tool was used by handlers to provide daily RPE records for their dogs.

After review and analysis of focus group discussions, the RPE logging tool was revised. The agility-related portion of the logging tool was revised to comprise only a single daily RPE for all agility-related activities. An indication of how much the weather conditions might have affected exertion for the dog on that date (none at all, a little, a moderate amount, a lot, a great deal) was added. A question related to mental or emotional exertion, stress, or stimulation was also added. Mental exertion was defined as sustained and prolonged cognitive (brain or mental) activity. Emotional exertion or stress was defined as a state of worry or mental tension caused by a difficult situation. Handlers provided a rating of mental or emotional stress or exertion on a scale in which 1 indicated no mental or emotional stress or exertion at all and 10 indicated the maximal possible mental and emotional exertion. The questions included in the revised RPE logging tool are shown in Figure 4.

### RPE logging

Between 12/6/2023 and 12/14/2023, handlers logged daily activities using the revised RPE logging tool. The number of days for which activity reports were logged for each dog/handler ranged from 4 to 8 days (mean = 6.9 days) for a total of 125 daily logging records. Of the 18 handlers, 14 (78%) submitted logging records for the requested 7 or 8 days. Median data entry time was 87 s (IQR = 56–117 s). Of the 125 logging records, one record had an incorrect date that indicated the information provided was for a date 4 days in the future. Of the remaining 124 logging records, 96 (77%) were entered on the day the activities were reported to have occurred, 20 records (16%) were entered on the day after the activities occurred, 6 records (5%) were entered 2 days after the activities occurred, and 2 records (2%) were entered 3 days after the activities occurred.

Handlers indicated that their dog engaged in some type of agility training or competition activity for 53 of 125 records (42%). Time spent in active agility training or competition activities for these dogs was defined as time in which the dog was actively working, excluding time that the dog may be resting between runs, while equipment is being moved, or while other dogs were working. The average time for active agility work for the 53 entries was 18.2 (SD = 10.5) minutes. An agility related RPE was provided for 52 of 53 (98%) logging entries. The median agility-related RPE for these dogs was 7 (IQR = 5–7, range = 2–10).

Of 125 logging records, 97 (77.6%) indicated that the dog had participated in one or more non-agility activities on the specified date (Table 2). An overall daily RPE was provided for 112 of 125 logging records (89.6%). The median overall RPE for all daily activities for all logging records was 4 (IQR = 3–6, range = 1–8).

**Table 2:** Frequency of indicated non-agility activities in 125 daily logging records.

Activity	Number	% (*n* = 125)
Running and playing alone or with other dogs	36	28.8%
Leash walk, less than 30 min	31	24.8%
No indication of other activities (no response)	28	22.4%
Leash walk, more than 30 min	26	20.8%
Core strength, balance, stretching, body awareness exercises	16	12.8%
Hiking, off leash, less than 30 min	13	10.4%
Trick training	12	9.6%
Hiking, off leash, more than 30 min	12	9.6%
Fetch activities (ball or disc)	8	6.4%
Obedience activities	6	4.8%
Nosework activities	2	1.6%
Lure coursing or Fast CAT activities	2	1.6%
Rally activities	1	0.8%
Herding or stock dog activities	1	0.8%
Swimming	0	0.0%
Flyball activities	0	0.0%
Dock jumping activities	0	0.0%
Barn hunt or earth dog activities	0	0.0%

All logging records included a response to the question as to whether or not the weather conditions had increased the dog’s exertion level for the day. Of the 125 responses, 69 (55.2%) indicated weather had no effect at all, 25 (20.0%) indicated it had a “little” effect, 14 (11.2%) indicated a “moderate” effect, 9 (7.2%) indicated that weather had “a lot” of effect, and 8 (6.4%) indicated that weather had a “great deal” of effect. Of the 125 logging records, 114 (91.2%) included a response to the question regarding mental exertion, stress, or stimulation on that date. The median stress rating was 4 (IQR = 3–6; range = 1–8).

### Follow-up questionnaire

Responses to the follow-up questionnaire were received from 13 of 18 handlers (72.2%). Handler ratings of the ease of use of the RPE logging tool had a bimodal distribution, which clustered between 1 and 3 (very easy) and between 8 and 10 (very hard) and had no response between the two peaks. The handlers who indicated higher ease of use scores (very hard) did not make any negative comments about the daily logging experience or the logging tool. The median estimated time for completion of the daily logging was 2 min (IQR = 2–5 min; range = 2–5 min). Every respondent except 1 stated that they preferred to do their logging at the end of each day or when they believed most activity for the day was concluded. All respondents except one stated that the email logging reminders were very helpful.

Most handlers expressed some level of confidence in the accuracy of their RPE ratings; some handlers stated that the ratings became easier with time as they developed their own internal calibration for their dog’s level of activities and stress. Two handlers felt that the overall daily RPE was harder to estimate than the agility related daily RPE. Only 3 handlers felt that completing the daily RPE logging record might have prompted them to modify their interactions with their dogs on that date. The question of how weather might impact exertion was raised by one handler, indicating that weather could have either a positive or negative effect and that wasn’t clear in the question. All respondents indicated that they thought the RPE logging tool was ready for wider use by larger numbers of handlers. Only one handler indicated that they thought a follow-up virtual meeting would be appropriate or necessary.

### Final RPE logging tool

**Figure 5:**
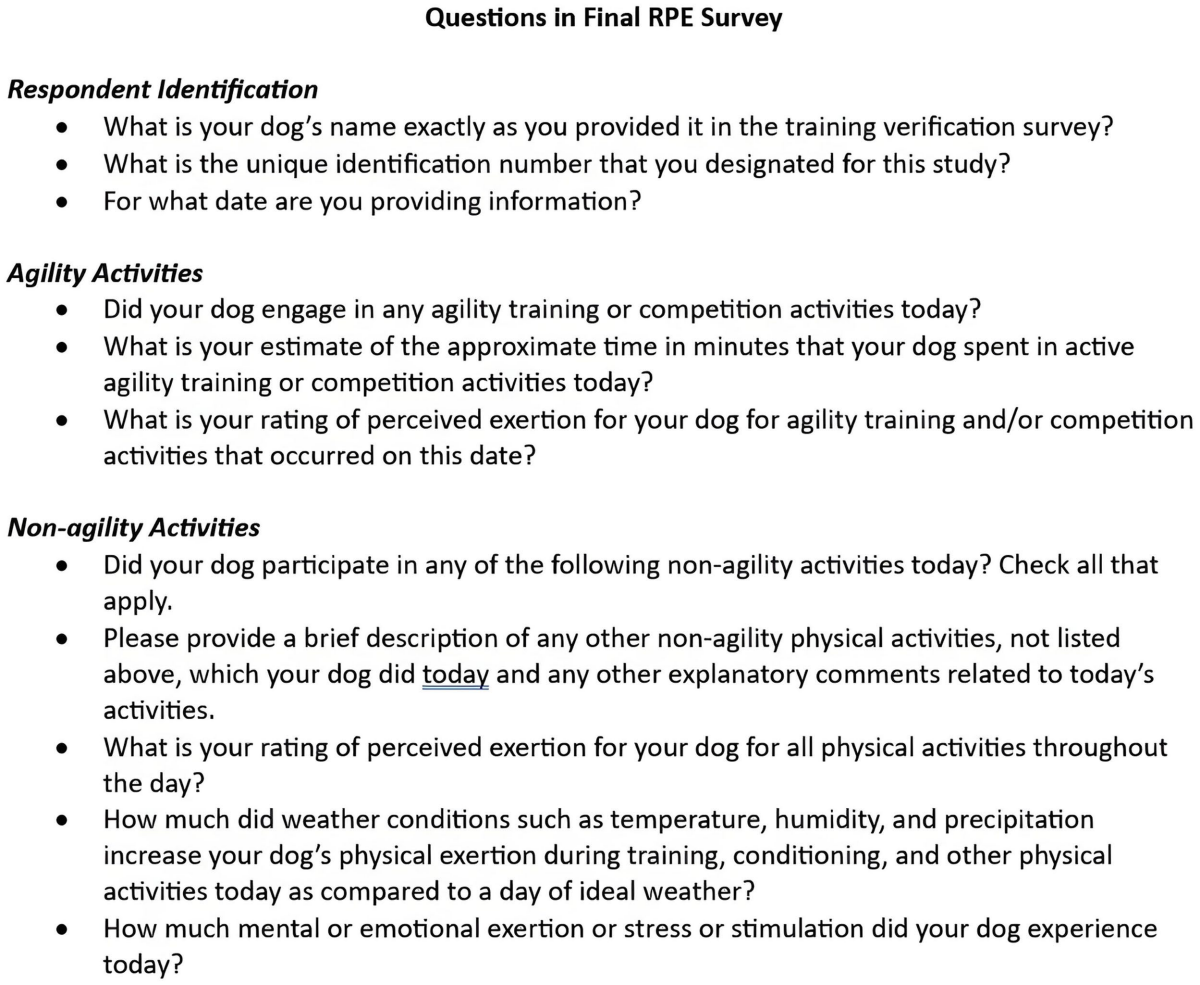
Final RPE logging tool with modifications based on handler feedback in follow-up questionnaire. This RPE logging tool will be used in future studies of agility dog training loads.

The research team reviewed all relevant data and made minor revisions to the logging tool. Changes were intended to further clarify individual questions. No substantive changes in number of questions, data requested, or type of question asked were made. The final version of the logging RPE tool is shown in Figure 5.

## Discussion

Monitoring athlete training load is considered critical to a science-based approach to training, fitness, and injury prevention. This report describes the development and initial evaluation of a tool that may be used by agility dog handlers to log daily activities quickly and easily and to report agility specific RPE and overall activity RPE as an aid in the measurement of training and activity load for their dog. The final RPE logging tool was developed in a six-step process that included: (1) initial drafting of an RPE logging tool by the research team; (2) review of the draft RPE logging tool with a group of US agility handlers using a semi-structured interview format and reflexive thematic analysis of their comments; (3) revision of the draft RPE logging tool by the research team; (4) seven days of activity logging by the same group of agility handlers using the revised RPE logging tool; (5) obtaining feedback from these handlers via online questionnaire; and (6) finalizing the RPE logging tool with consideration of all collected data.

The initial draft RPE logging tool was developed by the research team which included experienced researchers with deep knowledge of veterinary sports medicine and extensive personal experience in the sport of canine agility. The RPE scale as originally described by Borg ranged from 6 to 20 and was based on estimated human heart rate during exercise of 60 to 200 beats per minute (21). In the ensuing years, this scale has been adapted in a variety of ways for specific sports and user groups. For this canine RPE logging tool, a 10-point scale was used, similar to visual analog scales which are widely used for assessment of pain, and similar to modified RPE scales used for assessment of exercise intensity or training load in people and horses (15, 19). One previous description of a perceived exertion scale used for dogs on a treadmill used a 5-point scale ranging from 0, no effort noted, to 4, significant effort. For this report, the investigators chose to begin with the more common 10-point scale with the belief that agility dog handlers would be more knowledgeable of their dogs’ abilities and efforts than average dog owners or observers. As a result, it was theorized that agility handlers would be able to provide a more nuanced rating of their dog’s daily exertion.

The population demographics of the 18 handlers and dogs contributing to the data in this report were similar to what has been described for the overall population of agility handlers in the United States with a few notable differences. Handlers from the State of Washington were overrepresented in the group as compared to another recent study (10). This overrepresentation may have occurred because of prior acquaintance with the first author of this report. Handlers also tended to be younger than previously reported for the United States as a whole (10, 13), possibly because of greater familiarity and comfort with video-conferencing technology required for focus group interviews. Breed distributions were very similar to previous reports with border collies being most frequently included; one notable difference was the absence of mixed breed dogs in this report (32, 33). Despite these handler and dog demographic differences, the agility experiences of the dogs belonging to these handlers were very representative of the US agility population. Handlers reported competing with their dogs at all levels of competition from novice/beginner to excellent/masters and at jump heights from 8 to 24 inches. Approximately half of handlers had competed in at least one national competition in the past and their preferred venues for competition were diverse and similar to previous reports (10, 13, 32, 33).

Sample size calculation for qualitative interview or focus group studies is more nuanced than such calculations for quantitative research, but the estimates may be guided by concepts of “saturation” or “information power” (34–36). Code saturation is defined as the point at which no additional issues are identified in the data and meaning saturation is defined as the point at which no further insights or nuances are found (34). It is estimated that >80% of themes are captured within two to three focus group discussions and approximately 90% of themes within three to six focus groups (37). This coincides with an estimate of reaching code saturation within four group discussions and meaning saturation within five groups (34). The concept of information power proposes that smaller samples sizes are required for studies with a narrower aim, deep knowledge of the topic by study participants, a strong theoretical background to the study, strong quality of dialogue (often related to the research experience and skills of the interviewer), and an analytic strategy using in-depth analysis of narratives. All of these criteria were considered applicable to this project. As a result, considering the concepts of saturation and information power, it is concluded that the participant sample size and the number of focus groups were sufficient to collect the desired information for this project.

Analysis of data from focus group meetings was performed using the detailed contemporaneous notes of the first author. The lack of availability of video recordings or verbatim transcripts of the discussions may have decreased the richness of the qualitative analysis of the content of these meetings. Given the sample size (number of individuals and number of group sessions), however, it is likely that the most important codes and themes were identified. Reflexive thematic analysis is a common tool for analysis of qualitative psychological and sociological research data (31) but is rarely used in veterinary research. This analytical approach highlights the researcher’s active role in knowledge production through their engagement with the data and thematic conclusions (38). For the data analyzed in this report, it was a useful strategy to achieve the very narrow goal of optimizing the daily RPE logging tool for agility dogs. Through an iterative process of coding data and identifying themes, important insights into the clarity and ease of use of the RPE logging tool were identified and used to produce a revised draft tool. More importantly, this approach clearly identifies the need to develop online, accessible training options prior to wider implementation of the RPE logging tool and provides explicit suggestions and ideas for training content. These training materials should include both written and video options to maximize accessibility and ensure inclusive access to the information for diverse populations.

Revisions to the RPE logging tool included the addition of questions related to the effects of weather on agility related exertion and the effects of mental or emotional factors on overall exertion for the day. The addition of these questions was supported by results of reflexive thematic analysis and by evidence from human literature. Mental fatigue is well-documented to cause lowered performance in human athletes, with negative effects on technical and decision-making skills (39) and an association with greater perceived exertion (40). The overall level of life stress of the handler may also impact the dog’s performance in that long-term stress levels may be synchronized between dogs and handlers (41). Adverse weather conditions can greatly affect the amount of perceived exertion of athletes (42).

The observations that the average time for daily logging entry was <2 min and that nearly 95% of entries occurred within 1 calendar day of the events being logged support the general conclusion that the revised RPE logging tool was quick and easy to use by handlers in this study. The actual data logged provided interesting insights into agility training load. Fewer than half of the logging records indicated that the dog engaged in any specific agility-related activities during that logging day and more than 75% of records indicated that dogs engaged in a broad range of other types of athletic activities. Collectively, this strongly suggests that agility dogs promote and maintain athletic readiness through cross-training in other disciplines that can improve both physical and mental fitness. This is a hypothesis that should be further explored in more robust prospective studies.

In the final follow-up questionnaire, handlers expressed an overall high level of satisfaction with the RPE logging tool and its ease of use. Handlers expressed some confusion regarding the wording of the question related to weather effects on athletes. Comments in the follow-up questionnaire indicated that this could be interpreted as either a positive or a negative effect. The wording for this sentence was clarified in the final RPE logging tool. Other suggestions included in the responses to this final questionnaire largely related to requests for more sophisticated functionality including optimization of reminders, ability to review previous logging entries, and ability to produce summary reports. This functionality could easily be provided in a smartphone application with more flexible programming options than are available in the web-based software used for this project.

Overall, this work describes the process of development and initial testing of a daily RPE logging tool that may be used as an aid in the assessment of activity load in agility dogs. This approach and the resultant RPE logging tool could easily be adapted to monitor exertion levels in other types of canine athletes. Prior to widespread use, however, the daily RPE logging tool should be tested with a larger group of handlers and dogs over a longer period of time, work that is already in progress. In addition, validation of handler ratings should occur by comparison of RPE ratings with physiologic parameters such as heart rate or inertial measurement units (43, 44). Development of an application for use on mobile devices would allow for customization of preferences and utilization for a variety of canine athletes. Such customization should include direct access links to specific training videos and examples, automated integration with weather information, automated integration with heart rate or activity monitors, and customizable reminder and reward systems to improve consistency of daily logging. When fully validated and programmed as a smart-phone application, the agility RPE logging tool could be extremely valuable as a research tool for prospective studies of the effects of training load on performance and injury. Because of its ease of use and low cost, this tool will likely prove useful to individual handlers as an aid in planning, implementation, and monitoring of specific training and conditioning strategies.

## Data Availability

The raw data supporting the conclusions of this article will be made available by the authors, without undue reservation.

## Footnotes

^1^ www.qualtrics.com

^2^ www.zoom.us
