# Supplementary material for: Development of a web-based tool to assess daily rating of perceived exertion in agility dogs
Source: Front Vet Sci. 2024 Dec 6;11:1473977. doi: 10.3389/fvets.2024.1473977 (PMC11659274; doi:10.3389/fvets.2024.1473977)
Supplement: Supplementary file 1 [file Data_Sheet_1.pdf]

Default Question Block

Project Title: Agility dog activity load measurement using a web-based application and handler-provided rating of perceived exertion (RPE)
Principal Investigator: Debra C. Sellon, DVM, PhD
Contact Information: Email – canineagilityresearch@wsu.edu

Introduction

Purpose: There is very little information about the relationship between training load (duration and intensity of training activities) and risk of injury in agility dogs. In part, this is because we have very few ways to measure training load in canine athletes. In contrast, there is quite a lot of information on this topic for human and equine athletes. In this project we propose to perform preliminary testing of a very simple approach to quantifying training load in dogs.

This survey is an enrollment survey for Phase 1 of this study in which you can indicate your interest in helping with this research. We will review the information you provide about you and your dog, and we will let you know if you qualify for participation!

Eligibility: Participants must be 18 years of age or older and reside in the United States. You must be the handler of a specific dog which is currently competing with you as the handler in agility in the US. The dog may be competing in any US agility venue (e.g., AKC, ASCA, CPE, NADAC, UKI, USDAA) at any level. You and your dog should have no known injury or other circumstance that might impair normal activity. You must be willing to engage in

all study activities including viewing completing a short (< 5 min) daily activity survey for 5 - 7 days and participating in a follow-up virtual focus group discussions that will occur using Zoom or similar videoconferencing technology.

Participation: Participation in this study is completely voluntary. No details about yourself or your dogs will be used beyond what you choose to provide or directly approve in your answers. You may quit this project at any time and your information will not be included in our research if you quit before completion.

Estimated Time: This initial enrollment survey should take approximately 15 minutes to complete. If selected for participation, you will be asked to participate in an initial focus group discussion that will last approximately 30 to 60 minutes. For 5 - 7 consecutive days, you will be asked to complete a short (< 5 minutes) daily survey to document your dog's daily activities. At the conclusion of the test week, you will participate in a focus group discussion with other participants and members of the research team conducted via Zoom videoconferencing or similar technology. We anticipate that focus group sessions will require 30 – 60 minutes of your time. Based on your feedback, the survey instrument will be revised, and you may be asked to test it again followed by a second focus group discussion. This process will be repeated until a satisfactory RPE logging survey is developed, but the maximum number of testing and focus group cycles for any single participant will be two.

Who: The research team for this project includes Dr. Debra Sellon (Professor of Equine Medicine at Washington State University), Dr. Denis Marcellin-Little (Professor of Small Animal Orthopedic Surgery at University of California, Davis), Dr. Arielle Pechette Markley (Assistant Professor of Sports Medicine at The Ohio State University), Dr. Abigail Shoben (Associate Professor of Biostatistics at The Ohio State University), and Dr. Dianne McFarlane (Professor of Equine Medicine at University of Florida).

Benefits and Risks: The primary risk associated with participation in this

project is the risk of breach of confidentiality. We will collect your name and email address. This information will be stored in a password protected computer at Washington State University. Only the principal investigator for this project will have access to this information and this information will be deleted at the conclusion of the project. Data that is shared with investigators from institutions other than Washington State University will have identifiers (participant name and email address) removed. Upon enrollment, you will be a assigned a unique identification number. For daily logging surveys, you will use this unique identifying number and the name of your dog. For Zoom focus group meetings, you will not be asked or required to reveal your identity. We do not anticipate any other risks to you or your dog through your participation in this research. We are not asking you to modify any of your normal physical activities with your dog. We are only asking that you report those activities through a daily survey.

Ethics: This study was deemed exempt from review by the Institutional Review Board (IRB) at Washington State University. If you have any concerns about your rights as a participant, please contact the IRB at irb@wsu.edu . Continuing with this enrollment survey indicates your consent to participate in this project as it has been described on this page.

Funding: This study is funded by an Acorn grant from the American Kennel Club Canine Health Foundation.

If you have any questions, comments, or concerns, please contact Dr. Debra Sellon at canineagilityresearch@wsu.edu.

This section seeks to obtain information from you to confirm that you meet the inclusion criteria and are eligible to participate in this study.

Are you 18 years of age or older?

- ○ Yes

○ No

Do you currently reside in the United States?

○ Yes
○ No

Please provide your name and additional contact information as requested below.

	Please provide information in boxes below.
Your name	
Email address	
State of residence	

What is the name of the dog that you are nominating for enrollment in this project? This dog must be actively competing in agility in the US with you as their handler. The dog may be competing in any US agility venue (e.g., AKC, CPE, UKI, USDAA, NADAC) at any level.

At this time, does this dog or you as their agility handler have any known injury or other circumstance that might impair normal activity over the 4-week data collection period?

○ Yes
○ No

This section seeks to obtain information about the dog you have selected to participate in this project. Please answer the questions related to the specific dog you are nominating for enrollment.

What is this dog's date of birth?

____

How old is this dog in years?

____

What is the sex of this dog?

- ○ Intact male
- ○ Neutered male
- ○ Intact female
- ○ Spayed female
- ○ __________ Other, please specify

What is this dog's breed?

____

What is this dog's weight in pounds?

____

How would you classify your dog's weight? Reference the guide below in answering this question.

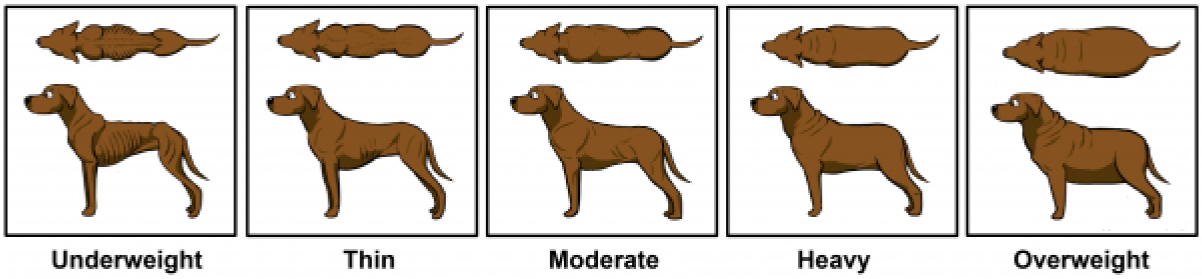

- ◯ Underweight (spine, pelvis, and ribs prominent from a distance; lack of muscle mass; hollow rump; obvious waist; thin neck)
- ◯ Thin (spine, pelvis, and ribs easily felt and visible on short-coated dogs; minimal body fat; obvious waist)
- ◯ Moderate, neither thin nor heavy (spine, pelvis, and ribs easily felt; last few ribs may be visible; rounded rump; tucked up waist)
- ◯ Heavy (spine, pelvis, and ribs felt but never visible; flat abdomen; dog appears square from the side and above)
- ◯ Overweight (spine, pelvis, and ribs difficult to feel; rolls of skin around neck and tail base; rounded abdomen; broad rump)

What is this dog's height in inches (measured at the withers)?

[ ]

In which agility venue is this dog most frequently competing at this time?

- ◯ American Kennel Club (AKC)
- ◯ Australian Shepherd Club of America (ASCA)
- ◯ Canine Performance Events (CPE)
- ◯ Dogs on Course in North America (DOCNA)
- ◯ North American Dog Agility Council (NADAC)
- ◯ Teacup Dogs Agility Association (TDAA)
- ◯ United States Dog Agility Association (USDAA)
- ◯ United Kingdom Agility International (UKI)
- ◯ United Kennel Club (UKC)
- ◯ [ ] Other, please specify

What is the highest level of agility at which this dog has competed in any venue?

- ◯ Starters/Novice/Beginner or equivalent
- ◯ Advanced/Open/Intermediate or equivalent
- ◯ Masters/Elite/Excellent or equivalent
- ◯ International
- ◯ ________ Other, please specify

What is your dog's approximate average speed in yards per second (yps) on a typical competition agility course containing only jumps, tunnels, and weave poles (no contact equipment)? If you are not sure, leave this question blank.

____

Has this dog ever competed at any national agility championship event in any venue?

- ◯ ________ Yes, please specify venue
- ◯ No

At what jump height does this dog most commonly compete in agility?

- ◯ 4 inches
- ◯ 8 inches
- ◯ 12 inches
- ◯ 16 inches
- ◯ 20 inches
- ◯ 24 inches
- ◯ ________ Other, please specify

On approximately how many ***days*** in the next year do you anticipate that this dog will likely compete in agility if handler and dog remain healthy and sound?

- 1 - 10 days
- 11 - 20 days
- 21 - 30 days
- 31 - 40 days
- 41 - 50 days
- 51 - 60 days
- > 60 days

You are almost finished! Please tell us some basic information about yourself.

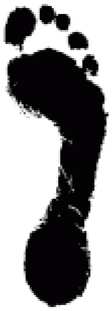

Approximately how many years have you been involved in agility training and competition activities?

Over your lifetime, with how many dogs have you ***competed*** in agility?

Have you ever competed at any agility national championship event with any

dog?

- Yes
- No
- ________ Other, please explain

Powered by Qualtrics
