# Supplementary material for: Development of a web-based tool to assess daily rating of perceived exertion in agility dogs
Source: Front Vet Sci. 2024 Dec 6;11:1473977. doi: 10.3389/fvets.2024.1473977 (PMC11659274; doi:10.3389/fvets.2024.1473977)
Supplement: Supplementary file 2 [file Data_Sheet_2.pdf]

Default Question Block

Project Title: Agility dog activity load measurement using a web-based application and handler-provided rating of perceived exertion (RPE)
Principal Investigator: Debra C. Sellon, DVM, PhD
Contact Information: Email – canineagilityresearch@wsu.edu

Purpose: There is very little information about the relationship between training load (duration and intensity of training activities) and risk of injury in agility dogs. In part, this is because we have very few ways to measure training load in canine athletes. In contrast, there is quite a lot of information on this topic for human and equine athletes. In this project we propose to perform preliminary testing of a very simple approach to quantifying training load in dogs.

Participation: Participation in this study is completely voluntary. No details about yourself or your dogs will be used beyond what you choose to provide or directly approve in your answers. You may quit this project at any time and your information will not be included in our research if you quit before completion.

Ethics: This study was deemed exempt from review by the Institutional Review Board (IRB) at Washington State University. If you have any concerns about your rights as a participant, please contact the IRB at irb@wsu.edu .

This survey is intended to obtain feedback and opinions from participants in Phase 1 of the study of agility rating of perceived exertion (RPE). Thank you for your assistance!

What is the name of the dog for which you were entering data in the daily logging survey?

Rate the ease of use of the daily logging survey on a scale of 1 (very easy) to 10 (very difficult).

	1	2	3	4	5	6	6	7	8	9	10	
Ease of use of the daily logging survey												

Approximately how long in minutes did it take you to complete the survey each day?

When did you most commonly input the responses for your dog?

- After I considered that most activity for the day had been concluded (same day)
- At the very end of the day (same day)
- The next morning
- At a later time on the day after the exercise occurred
- More than a day after the exercise occurred
- Other, please explain or comment in the next question

Do you have any comments about the time of day you typically found it most convenient or best to provide your RPE logging data?

Were the daily email reminders helpful to you?

- Yes, very helpful
- Somewhat helpful
- Not really necessary or helpful
- Annoying
- What email reminders?

Do you have any additional comments about the email reminders? These reminders were sent at 12 pm (noon) Pacific time. Was this a reasonable time for the reminders or do you have another suggestion?

Do you feel that you were able to provide a reasonably accurate rating of perceived exertion for your dog for agility-related activities on each day? Please provide comments as appropriate.

Do you have suggestions for other non-agility activities that should be added to the list that is currently used in the RPE logging survey?

Do you feel that you were able to provide a reasonably accurate rating of perceived exertion for your dog for all physical activities on each day? Please provide comments as appropriate.

Do you feel that you modified your daily interactions with your dog in any way because of the anticipation of providing RPE ratings on that day?

- Definitely not
- Probably not
- Might or might not
- Probably yes
- Definitely yes

Please provide comments or explanations about how the use of the daily RPE logging survey may have impacted your daily interactions with your dog.

The questions on the effect of weather on your dog's exertion and the amount of mental or emotional exertion/stress/stimulation your dog experienced were added to the daily RPE survey based on suggestions from Phase 1 participants. What was your reaction to those questions? Do

you feel they were helpful? Were they relatively easy for you to answer?

Are there specific changes to the daily RPE logging survey that you feel should be made prior to more widespread use of the daily survey in Phase 2 of this project?

Do you have additional comments about the daily RPE logging survey you would like to provide? Do you think this project is ready to move forward into Phase 2? Would you recommend Phase 2 participation to your friends and acquaintances?

Would you like the opportunity to have a Zoom meeting with representatives from the research team to discuss your experiences further?

- Yes. I definitely think we need to do this.
- Probably not necessary, but I would likely participate if requested.
- I don't think another Zoom meeting is necessary at this time. I feel I have provided all the input and insights that I can at this time!

Powered by Qualtrics
